# Motor Inhibition during Overt and Covert Actions: An Electrical Neuroimaging Study

**DOI:** 10.1371/journal.pone.0126800

**Published:** 2015-05-22

**Authors:** Monica Angelini, Marta Calbi, Annachiara Ferrari, Beatrice Sbriscia-Fioretti, Michele Franca, Vittorio Gallese, Maria Alessandra Umiltà

**Affiliations:** 1 Department of Neuroscience, Unit of Physiology, University of Parma, Parma, Italy; 2 Department of Neuroscience, Unit of Child Neuropsychiatry, University of Parma, Parma, Italy; 3 Department of Pharmacy, University of Parma, Parma, Italy; University of Rome, ITALY

## Abstract

Given ample evidence for shared cortical structures involved in encoding actions, whether or not subsequently executed, a still unsolved problem is the identification of neural mechanisms of motor inhibition, preventing “covert actions” as motor imagery from being performed, in spite of the activation of the motor system. The principal aims of the present study were the evaluation of: 1) the presence in covert actions as motor imagery of putative motor inhibitory mechanisms; 2) their underlying cerebral sources; 3) their differences or similarities with respect to cerebral networks underpinning the inhibition of overt actions during a Go/NoGo task. For these purposes, we performed a high density EEG study evaluating the cerebral microstates and their related sources elicited during two types of Go/NoGo tasks, requiring the execution or withholding of an overt or a covert imagined action, respectively. Our results show for the first time the engagement during motor imagery of key nodes of a putative inhibitory network (including pre-supplementary motor area and right inferior frontal gyrus) partially overlapping with those activated for the inhibition of an overt action during the overt NoGo condition. At the same time, different patterns of temporal recruitment in these shared neural inhibitory substrates are shown, in accord with the intended overt or covert modality of action performance. The evidence that apparently divergent mechanisms such as controlled inhibition of overt actions and contingent automatic inhibition of covert actions do indeed share partially overlapping neural substrates, further challenges the rigid dichotomy between conscious, explicit, flexible and unconscious, implicit, inflexible forms of motor behavioral control.

## Introduction

Motor imagery (MI) is the conscious, voluntary rehearsal of action representations without any overt movement [1]. According to the “motor simulation theory” proposed by Marc Jeannerod [2] common neural substrates underlie both pre-movement phase of executed actions (overt actions) and potential motor acts (covert actions) like MI. Supporting this hypothesis, to date a growing number of human functional neuroimaging studies have shown during MI and Action Execution (AE) a substantial, even if incomplete, overlap of active motor-related brain regions, including frontal premotor, parietal and subcortical regions [3–5].

Given this ample evidence for a shared set of cerebral regions involved in encoding actions, whether or not those actions are effectively executed, a still unsolved problem is the identification of the neural mechanisms of motor inhibition, preventing covert actions from being performed and, consequently, allowing them to remain “potential”, without overt movements, in spite of the activation of the motor system.

Two principal mechanisms of motor inhibition have been proposed [6]. The first one acts at the cortical level, preventing the motor programs elaborated within the parieto-premotor circuits from activating the primary motor cortex (M1). In this regard, the pre-supplementary motor area (pre-SMA) is thought to be part of a crucial motor inhibitory network, including the right inferior frontal gyrus (rIFG) and the basal ganglia (BG) [7]: through the involvement of the subthalamic nucleus (STN) and the hyperdirect pathway or the striatum and the indirect pathway, these frontal areas would generate downstream inhibitory effects on facilitatory thalamo-cortical output directed to M1.

The second hypothesized mechanism consists of the inhibition of the descending motor command before it reaches the motoneuronal level, through inhibitory or disfacilitatory influences at the spinal level [2]. Premotor areas as ventral premotor cortex (vPMC) in the IFG and dorsal premotor cortex (dPMC) could play a relevant role in the control of spinal circuits, by means of their spinal projections, direct or indirect through the brainstem [8]; at the same time, these areas could also act at a cortical level through direct connections with M1, exerting suppression of its excitatory output.

To date, how motor inhibition is enacted during MI and which cerebral networks underpin such inhibition remain open questions. In particular, could cerebral regions (such as the pre-SMA and the rIFG), thought to be involved in the inhibition of overt actions [9], also represent the cerebral substrates of the inhibition put into action during MI? A typical paradigm used to test the inhibitory control of overt actions is the Go/NoGo task, eliciting two event-related potentials (ERPs) associated with NoGo trials, interpreted as electrophysiological markers of inhibition: 1) the NoGo-N2, a negative deflection with larger amplitude during NoGo relative to Go trials, with a fronto-central scalp distribution and a latency of 200–400 ms post-stimulus onset [10]; 2) the NoGo-P3, an enhanced positive deflection with maximum at Fz and Cz in NoGo relative to Go trials [11] and a latency of 300–500 ms post-stimulus onset. The functional meaning of these ERPs is, however, still debated: it has been suggested that NoGo-N2 could better reflect an early non-motoric stage of inhibition, or a process of conflict monitoring between incompatible task responses for the focusing of top-down attentional control [12–14]. Similarly, the NoGo-P3 is considered too late to reflect an ongoing inhibitory operation, peaking at or even after the overt response [12]; alternatively, it has been associated with an evaluative processing of the outcome of inhibition [for reviews see: 15, 16].

Likely, multiple parallel operations are engaged during the NoGo-N2 and NoGo-P3 time windows [17, 18], since the Go/NoGo task requires not only inhibition but also decision making, response selection and planning. This could explain the conflicting results regarding functional meaning and source generators of the NoGo-N2 and NoGo-P3 waves, as well as the large number of cerebral regions shown by fMRI studies of Go/NoGo task [for reviews see: 16, 19]: dominant sources for the NoGo-N2 were found in bilateral prefrontal cortex [20], in the anterior cingulate cortex (ACC) [13], in right ventrolateral and dorsolateral prefrontal cortex (DLPFC) [21]. Generators of the NoGo-P3 were reported in the right frontal lobe [22], but also in orbitofrontal cortex [23], in ACC and left premotor cortex [20]. fMRI studies revealed activations in multiple cortical and subcortical regions, including the pre-SMA, the rIFG, the BG, but also the DLPFC, the dPMC, the inferior parietal lobule (IPL), the ACC [16, 19]. Probably, many of these regions are not involved directly in the inhibitory commands, but rather in different concomitant cognitive processes [16, 19].

Of note, fMRI could not well describe the exact timeline of activations and the dynamic interaction between different brain areas in real time, due to its low temporal resolution [24]. Furthermore, most of Go/NoGo ERP studies used a traditional waveform analysis: such canonical approach is based on the description of ERPs in terms of waves with peaks and troughs and on the assessment of the amplitude and latency of such components. This approach introduces a degree of experimental bias, related to the *a priori* selection of scalp sites and time periods to optimally evaluate the predefined ERP components of interest [25, 26]. Additionally, ERP waveform analysis could not accurately reflect and define temporally overlapping activities of the different neural subsystems involved in ERP generation.

In order to avoid these methodological limitations, which could be at least partially responsible for the inconsistent source localizations and functional interpretations of NoGo-N2 and NoGo-P3 waveforms [25, 27], more recently new data-driven methods of decomposition of multichannel scalp field data have been applied to Go/NoGo and Stop-Signal tasks [e.g., 17, 18, 28, 29]. The present study is based on a spatio-temporal analysis of the scalp electric field using the “microstates segmentation approach” [27]. This approach summarizes ERP data in a sequence of time periods of stable scalp topography, called “segmentation maps”, thought to represent “functional microstates” of the brain (i.e., discrete computational steps during information processing) [30]. These periods of stable scalp topography are a more objective means for defining ERP components and time windows for source analysis, relying on the statistical proof that the electric fields are different and thus generated by different neural sources.

Taking into account this background, we performed a high density EEG study evaluating the cerebral microstates and their related sources elicited during two types of Go/NoGo task, requiring the execution or withholding of an overt (Go) or a covert (MI) action, respectively. The preliminary assumption of our study was that the covert MI condition would elicit not only voluntarily evoked motor representations, but also a parallel inhibitory mechanism, whose cerebral substrates could be possibly similar to those elicited for inhibitory control of overt actions during an overt NoGo condition, and presumably overlapping with NoGo-N2 and/or NoGo-P3 latency time ranges reported in the literature.

Hence, the principal aims of the present study were the evaluation of: 1) the presence during MI of putative motor inhibitory mechanisms; 2) their underlying cerebral sources; 3) their differences or similarities with respect to cerebral networks underpinning the inhibitory control of overt actions during the overt NoGo condition. Furthermore, our results could also contribute to clarify some of the controversies emerged from previous Go/NoGo functional neuroimaging and EEG studies, taking advantage, on the one hand, of the higher temporal resolution of high-density EEG technique and, on the other, of the use of microstates approach with respect to canonical raw voltages analysis.

## Materials and Methods

### Participants

Twenty-one participants were initially recruited for the study; six participants with bad EEG signals or with less than 40 artifact-free correct trials per condition were subsequently discarded, resulting in a final sample of 15 young adult volunteers: 9 males, 6 females; mean age ± standard deviation (SD): 24.4 ± 3.8 years; age range: 20–35 years. All participants had normal or corrected-to-normal visual acuity, no history of psychiatric or neurological impairments and were right-handed, as assessed by the Edinburgh Handedness Inventory [31]. All participants provided a written informed consent to participate in the study, which has been approved by the local ethical committee (Comitato Etico per Parma) and has been conducted according to the principles expressed in the Declaration of Helsinki.

### Stimuli and Procedure

The experimental paradigm was a modified form of the cued O-X Continuous Performance Task (CPT), already used in previous Go/NoGo studies [e.g., 11, 22, 32, 33]. From the initial development [34], several CPT versions have been extensively used to assess executive functions, in particular sustained and selective attention and response inhibition [11, 22, 32, 33, 35, 36]. In cued forms of CPT, interspersed among a large number of distractors, a warning signal (cue) precedes imperative signals (targets) that require the execution (Go condition) or the withholding (NoGo condition) of a motor response. Hence, cued forms of CPT are suitable for the evaluation of the cognitive control of the motor system, requiring at target onset a decision-making process between executing or refraining from an anticipated motor response [for a comparison between the CPT and Go/NoGo task see: 36]. In particular, the cued O-X CPT, similar to that used in the current study, has been proved to be a powerful tool to investigate motor inhibitory control [11, 22, 32, 33].

**Fig 1:**
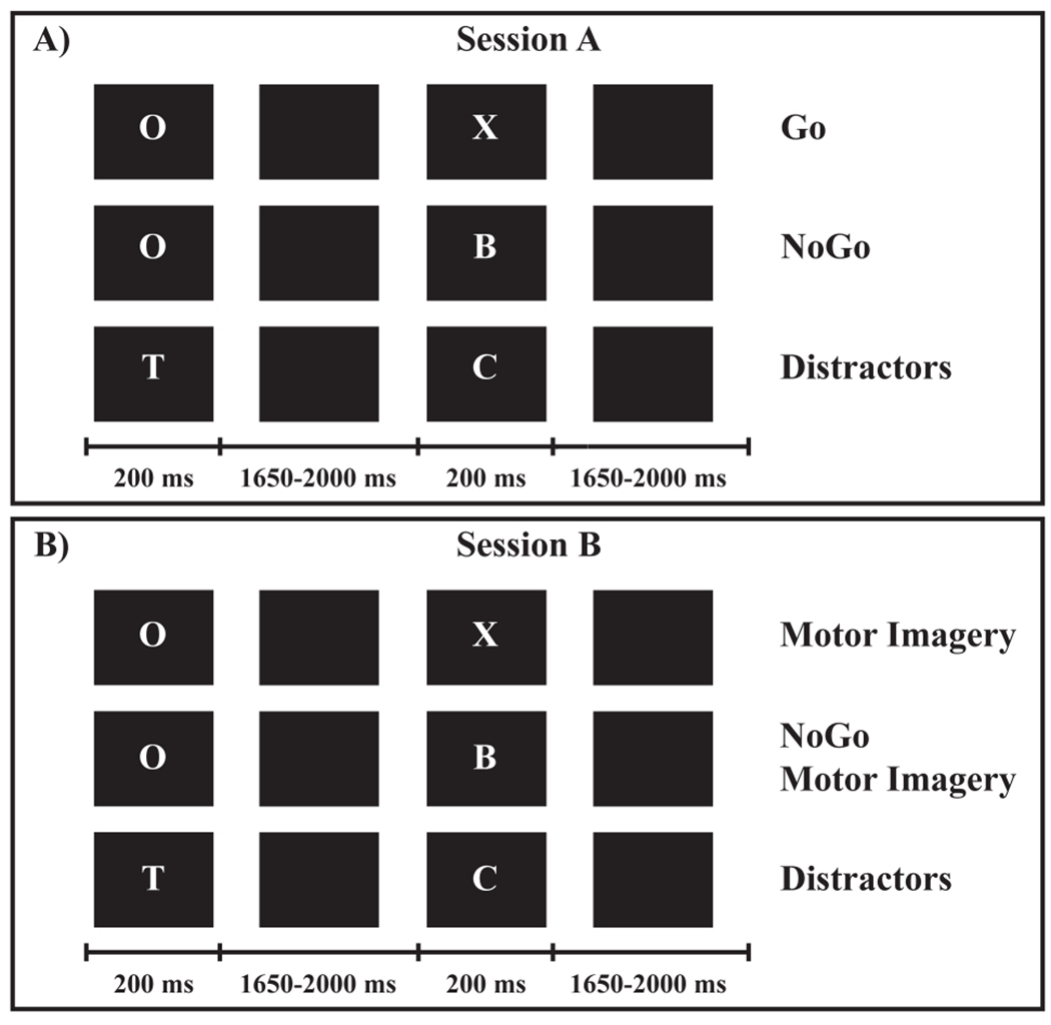
Experimental paradigm and stimuli. **(A)** Session A: Go and NoGo conditions. (**B)** Session B: Motor Imagery and NoGo Motor Imagery conditions.

Our paradigm consisted of four conditions organized in two blocks (sessions A, B) (Fig 1A and 1B): Go and NoGo conditions were presented in session A; MI and NoGo Motor Imagery (NoGoMI) conditions were tested in session B. We separated the four conditions in two blocks in order to maintain a clear distinction between the controlled inhibitory mechanism in NoGo condition and the putative motor inhibition in MI condition, and to avoid potentially confounding interferences leading to difficult interpretations of the results. The order of presentation of the two sessions was balanced among participants.

Stimuli consisted of 12 different white letters (A-H, J, L, O and X) on a black background, presented sequentially in pseudo-random order at the center of a 19-inches computer screen positioned at 60 cm from participants. The letters on the screen were 20 mm high and 15 mm wide, resulting in a visual angle of 1.91° vertically and 1.43° horizontally. The same letter was never immediately repeated. Each letter was presented for 200 ms and separated from the next one by a black screen whose duration varied randomly between 1650 and 2000 ms, in order to minimize the temporal predictability of stimuli appearance [37]. In both sessions (Fig 1) the letter “O” was the preparatory cue, followed by the imperative target stimulus which specified the requested responses. In session A (Fig 1A) the letter “X” after the “O” cue represented the target stimulus for Go condition, requesting the execution of a button press (see below). In session B (Fig 1B) the letter “X” after the “O” cue constituted the target stimulus for the MI condition, requesting the kinesthetic MI of the button-press movement (see below). In both sessions the other letters (A–H, J, L) required to refrain from responding if they immediately followed an “O”, representing target stimuli in session A for NoGo and in session B for NoGoMI conditions, respectively. Conversely, they served as meaningless distractors if not preceded by an “O”.

Each of the two sessions consisted of 80 trials “O-X” (Go and MI trials, in session A and B respectively), 80 trials “O-noX” (NoGo and NoGoMI, in session A and B respectively) and 240 distractors. The sequence of presentation of trials and distractors was randomized. Each session lasted about 20 minutes, with a 5 minutes rest period between the two sessions.

Before starting the recording, participants completed a brief training phase. They were presented with a training block including two parts (A and B), whose order of presentation was balanced among participants: each part included 4 trials for each condition (4 Go and 4 NoGo trials in part A; 4 MI and 4 NoGoMI trials in part B) and 16 distractors. Participants familiarized with the Go motor task (consisting in pressing a button on a pad, positioned in front of them, with the index finger of their right hand) and with the MI task. For the MI task, participants were specifically instructed to imagine themselves pressing the button in a first-person perspective, i.e., to perform a kinesthetic MI and not only to visually imagine the movement. Speed and accuracy in motor responses were emphasized equally during the explanation of the tasks. The instructions given for the Go/NoGo and MI/NoGoMI tasks were maintained uniform between the two sessions (i.e.: for the Go and MI conditions participants were requested to press or to imagine themselves pressing the button, respectively; for the NoGo and the NoGoMI conditions participants were requested to refrain from responding or to imagine themselves refraining from responding, respectively). For the NoGoMI condition, participants were not explicitly asked to actively think to suppress an imagined button-press movement.

Stimuli delivery and response recording were controlled with the E-prime 2.0 software; the button-press recording was used to assess omission (i.e., Go trials without responses) and commission (i.e., responses in NoGo trials) errors.

### EEG Recording and Preprocessing

Continuous EEG was recorded using the 128-channels Geodesic EEG System (Electrical Geodesics Inc., Oregon) and the HydroCel Geodesic Sensor Net (GSN300), at a sampling rate of 500 Hz (0.01 Hz high-pass filter) with the vertex as on-line reference; electrodes impedances were kept below 50 kΩ. Off-line analyses were performed with Cartool software (freely available at: http://brainmapping.unige.ch/cartool) [26]. The raw EEG data were band-pass filtered (1–30 Hz, notch 50 Hz) and recalculated against the average reference.

Since the principal aim of the current study was to assess whether a putative inhibitory mechanism was engaged during MI enactment, we focused EEG analyses on cerebral activities elicited after the appearance of the target signals.

Epochs from 200 ms pre-target onset to 700 ms post-target onset were averaged across trials, separately for each participant and condition; these single-subject averages were then used to compute four group-averaged ERPs, one for each condition. Trials with incorrect responses (omission and commission errors) and NoGo, MI and NoGoMI trials with concomitant EMG activity were excluded (see: EMG recording). The remaining trials were submitted to an automated threshold rejection criterion of 65 μV and visually inspected for detection of ocular, muscular and other artifacts. To maintain a good signal-to-noise ratio, a lower limit of 40 artifact-free correct trials per participant per condition was set. The mean ± SD of accepted epochs was: for Go condition 45.3 ± 1.45; for NoGo condition 44.7 ± 2.9; for MI condition 45.1 ± 1.8; for NoGoMI condition 43.8 ± 3.6. A repeated measures ANOVA (P < 0.05), performed in order to exclude differences in the number of accepted trials among conditions, did not result significant (F _(3,42)_ = 2, P > 0.05). The outermost belt of electrodes of the sensor net, more prone to show residual muscular artifacts, was excluded and the original template was reduced from 128 to 110 channels. Artifacted channels were interpolated using a spherical spline interpolation method implemented in the Cartool software [38].

### EEG Microstate Analysis

The first 700 ms post-target period, containing NoGo-N2 and NoGo-P3 ERPs described in literature [16], were analyzed in terms of the spatio-temporal characteristics of the global electric field on the scalp [25–27]. A pattern analysis of the ERP scalp topography based on a modified hierarchical clustering algorithm termed “Atomize and Agglomerate Hierarchical Clustering” [25] was performed on the group-averaged ERPs, in order to summarize data by a limited number of scalp potential fields (“segmentation maps” or “microstates”), and to identify their sequence over time within a given dataset. This cluster analysis is reference-free and insensitive to pure amplitude modulation of the same scalp potential field across conditions, since normalized maps are compared. It was performed across time and experimental conditions separately for the two sessions: one segmentation procedure was applied on Go and NoGo conditions data, and another one on MI and NoGoMI data. A temporal criterion of a minimal duration of a given map in the group-averaged data for at least 10 consecutive data points (20 ms at our 500 Hz sampling rate) was applied [26]. The optimal number of maps (i.e., the minimal number of maps accounting for the greatest variance of the dataset) was assessed by a modified Krzanowski-Lai (KL) criterion [25, 39].

The pattern of maps resulting from the cluster analysis performed on the group-averaged dataset, was statistically tested at the level of the ERPs of each participant, by means of the “single-subject fitting” procedure. This competitive fitting procedure is based on the calculation of the strength-independent spatial correlation between single-subject ERPs and each segmentation map identified in the group-averaged data [24, 25]. Each time point of the single-subject ERPs was labeled according to the selected map with which it best correlated spatially: the output of the fitting is a measure of the relative “map presence” (i.e., the number of ms of the single-subject ERPs for each condition that are assigned to the specific map, resulting from cluster analysis performed on group-averaged data, with which they correlated best). If different maps appeared in a given time window in different conditions, repeated measures ANOVAs (P < 0.05), with Condition and Map as within-subject factors, were performed on the map presence data resulting from the single-subject fitting. Any significant factors interaction was further evaluated by means of planned comparisons (P < 0.05). The cluster and fitting analyses determined whether and when different experimental conditions were more often described by one map versus another, and therefore if different neural generators better accounted for particular experimental conditions [24, 25].

### Source Analysis

As a final step, the electrical source analysis of the segmentation maps was performed, using a distributed linear inverse solution based on a Local Auto-Regressive Average (LAURA) regularization approach [40]. LAURA model reconstructs the brain electric activity in each point of a 3D grid of solution points, selecting the source configuration that better mimics the biophysical behavior of electric fields without *a priori* assumption on the number of dipoles in the brain. The solution space was calculated on a locally spherical head model with anatomical constraints (L-SMAC) [41] and comprised 3001 solution points (voxels) homogeneously distributed within the brain structures of the Montreal Neurological Institute (MNI152) average brain. All solution points were labeled with their Talairach and Tournoux coordinates [42] as well as their anatomical labels.

As a preliminary step, the source of each mean segmentation map was evaluated, applying the LAURA algorithm at the group-averaged ERP fields of the four conditions. This operation does not give indications about the statistical reliability of these sources at the individual level and provides only one current density maximum for each segmentation map: consequently, weak but consistent differences in other areas could be ignored due to thresholding. Hence, to statistically validate whether these distributed activations over all solution points were significantly different among conditions, we conducted a “voxel-wise parametric mapping analysis” at individual level [27]: when different maps were present among conditions, paired t-tests were performed for each solution point. To do that, individual ERP data were averaged over the time period of each different map, in order to generate a single data point per period for each participant and condition. The LAURA current densities source estimations for each solution point were then contrasted by means of paired t-tests. These statistical comparisons were performed first between conditions in each session and then by contrasting MI with Go and with NoGo conditions data. Solution points with P-values < 0.05 (t _(14)_ > 2.14/ < -2.14) were considered significant; in addition, a cluster threshold of at least 10 contiguous activated solution points was applied. Source analyses were performed with Cartool software (http://brainmapping.unige.ch/cartool).

### EMG Recording and Analysis

Surface EMG of the right First Dorsal Interosseous (FDI) muscle was recorded with EGI’s Polygraph Input Box (PIB) continuously during both experimental sessions (sampling rate 500 Hz, band-pass filter 30–200 Hz, notch 50 Hz) using bipolar derivation. A moving average (period = 300 ms), centered on each 100 ms epoch, was applied to the rectified EMG data of each participant recorded in the time interval from -200 (baseline) to 700 ms from target stimulus onset. An offset procedure was performed using as offset value the mean baseline EMG plus its standard deviation multiplied by two (baseline threshold). This latter value was compared, by means of independent samples t-test with a significance criterion of P < 0.01, with the baseline. The aim of EMG recording was twofold. Firstly, in order to control for the possibility that differences in EEG activity among conditions could have been influenced by residual movements, NoGo, MI and NoGoMI trials with significant EMG activity, identified with the procedure described above, were discarded.

Secondly, since the electrophysiological correlates of motor inhibition need to be present before the movement onset, in order to define the temporal relationship between cortical activity and the motor response, in Go trials the mean latency of the first rising phase of the EMG activity, measured with respect to the onset of the Go stimulus (Go EMG onset), was calculated.

### Motor Imagery Assessment

After the EEG recording, the MI ability of participants was evaluated by means of the Vividness of Movement Imagery Questionnaire (VMIQ) [43]. The VMIQ consists of 24 items, each of which is a description of a common movement (e.g., walking, kicking a ball in the air). Participants were asked to imagine each item from a third-person (external imagery) and from a first-person (internal imagery) perspective: then, they rated the vividness of the imagined movement on a 5-point Likert-type scale, with responses ranging from 1 (perfectly clear image) to 5 (no image at all). The rating procedure for the questionnaire is summative, with the lower score indexing a more vivid imagery. Three scores were obtained: 1) VMIQ-Other (range 24–120), for the external imagery; 2) VMIQ-Self (range 24–120), for the internal imagery; 3) VMIQ-Total (range 24–240), resulting from the addition of the other two scores.

## Results

### Performance and EMG Recording

The mean percentage of incorrect responses ± SD for Go condition (omission errors) was 2.08% ± 2.52, and for NoGo condition (commission errors) was 2.25% ± 2.72.

The mean ± SD Go EMG onset was 415 ± 69 ms after Go target presentation.

### Motor Imagery Assessment

The mean VMIQ-Total score ± SD was 103.4 ± 25.24; the mean VMIQ-Other score ± SD was 52.07 ± 14.3; the mean VMIQ-Self score ± SD was 51.33 ± 14.52. On average, participants had “clear and reasonably vivid” external (mean score on the 5-point Likert-type scale ± SD: 2.17 ± 1.05) and internal (mean ± SD: 2.14 ± 1.13) MI ability.

### EEG Microstate Analysis

**Fig 2:**
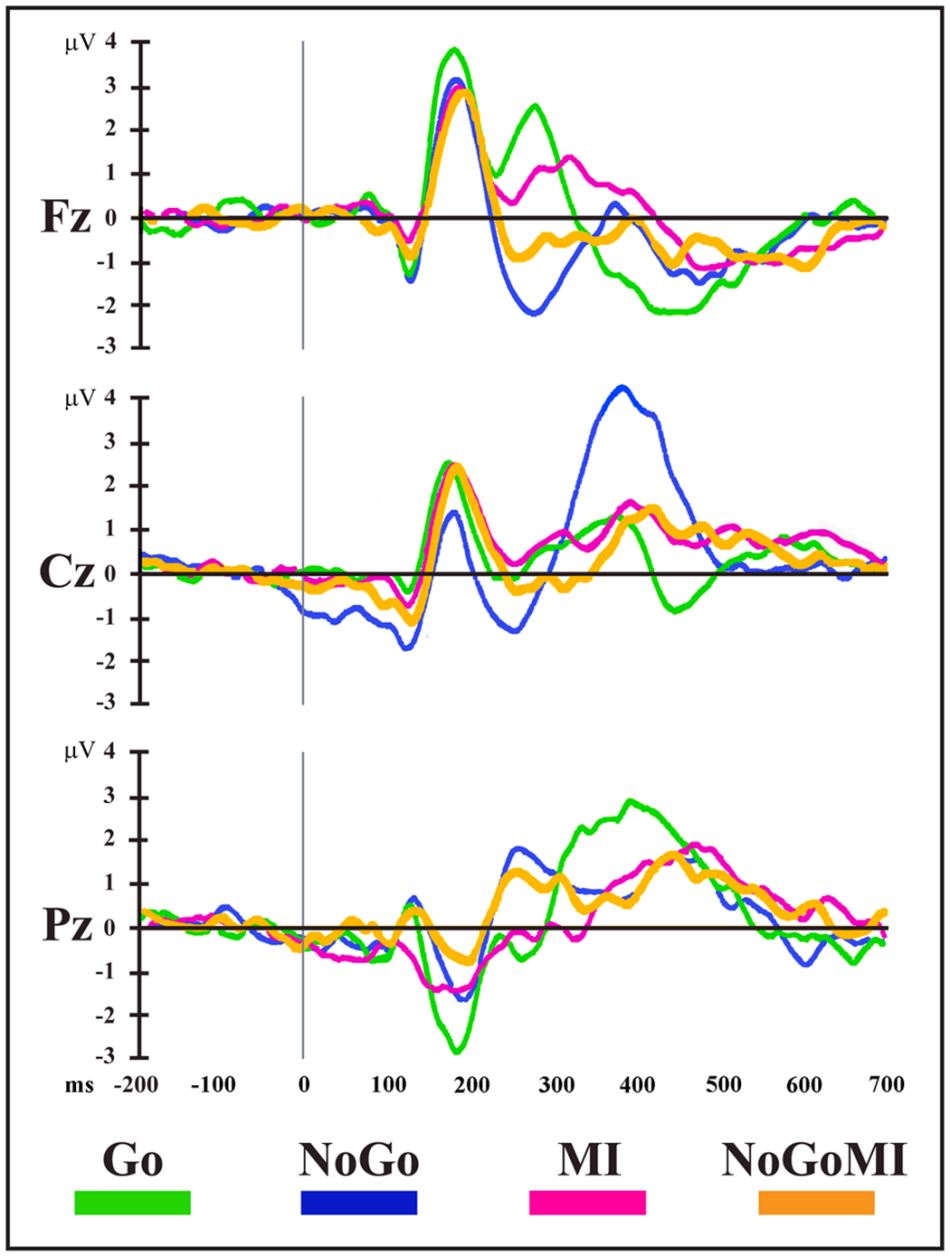
Event related potential (ERP) waveforms. Group-averaged (n = 15) stimulus-locked ERP waveforms (plotted as voltage in μV in function of time in ms, stimulus onset: 0 ms) for the four experimental conditions from Fz, Cz and Pz electrodes. MI: Motor Imagery; NoGoMI: NoGo Motor Imagery.

For completeness and to allow comparison of the results of microstate analysis to previous literature on Go/NoGo tasks based on ERP waveform analysis, the superimposed group-averaged (n = 15) ERP waveforms of the four conditions from selected midline electrodes, where maximum modulatory effects are expected [10–16], are shown in Fig 2. Of note, the typical N2 and P3 Go/NoGo effects were replicated. By visual inspection, a clear NoGo-N2 (peak at Fz, with amplitude: -2.1 μV and latency: 276 ms) and NoGo-P3 component (peak at Cz, with amplitude: 4.21 μV and latency: 382 ms) emerged.

**Fig 3:**
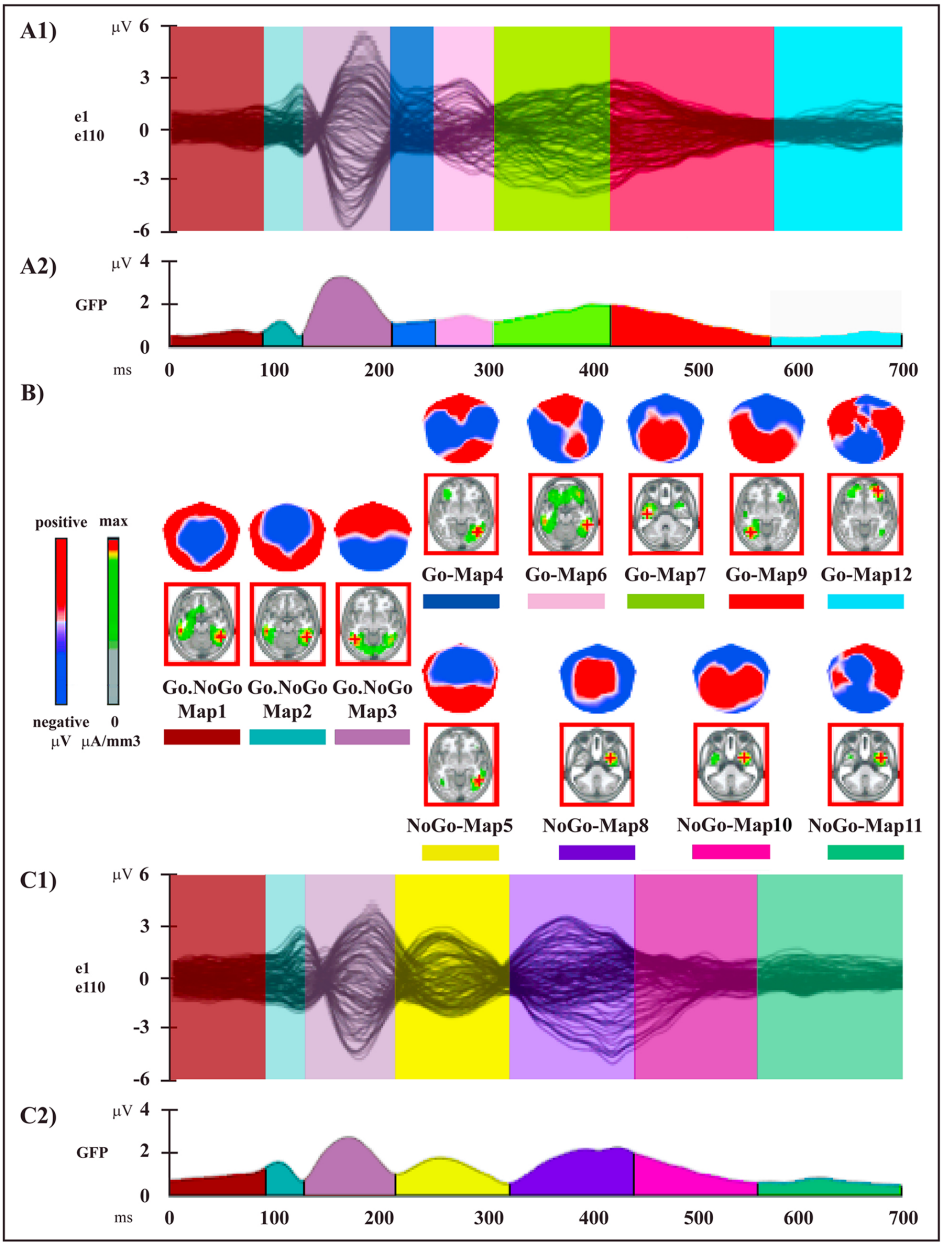
Electrophysiological results over the 700 ms post-stimulus period (stimulus onset: 0 ms) of session A. **(A1** and **C1)** Group-averaged (n = 15) ERP waveforms for Go (A1) and NoGo (C1) conditions, superimposed across the 110 recording channels (e1–e110). **(A2** and **C2)** Microstate segmentation results for Go (A2) and NoGo (C2) conditions. The temporal distribution of the microstates in each condition revealed by the spatio-temporal segmentation analysis applied on session A dataset is reported on the curve of the global field power (GFP) (i.e., the variance of the 110 channels over the whole scalp at a given time point). Each microstate and its temporal window are indicated by different colors; the same color indicates the same microstate. **(B)** Mean topographic maps and related mean LAURA source estimations (in red panels) corresponding to each microstate for the group-averaged ERP data. All topographic maps are plotted with nasion upward and left scalp leftward; each map is scaled separately with respect to its maximum and minimum values to optimise the contrast. The current density maxima resulting from source estimations (green: low current density; red: high current density) are rendered on horizontal slices of MNI152 template brain (left hemisphere on the left side); source estimation for each microstate is independently scaled with respect to its maximum value.

**Fig 4:**
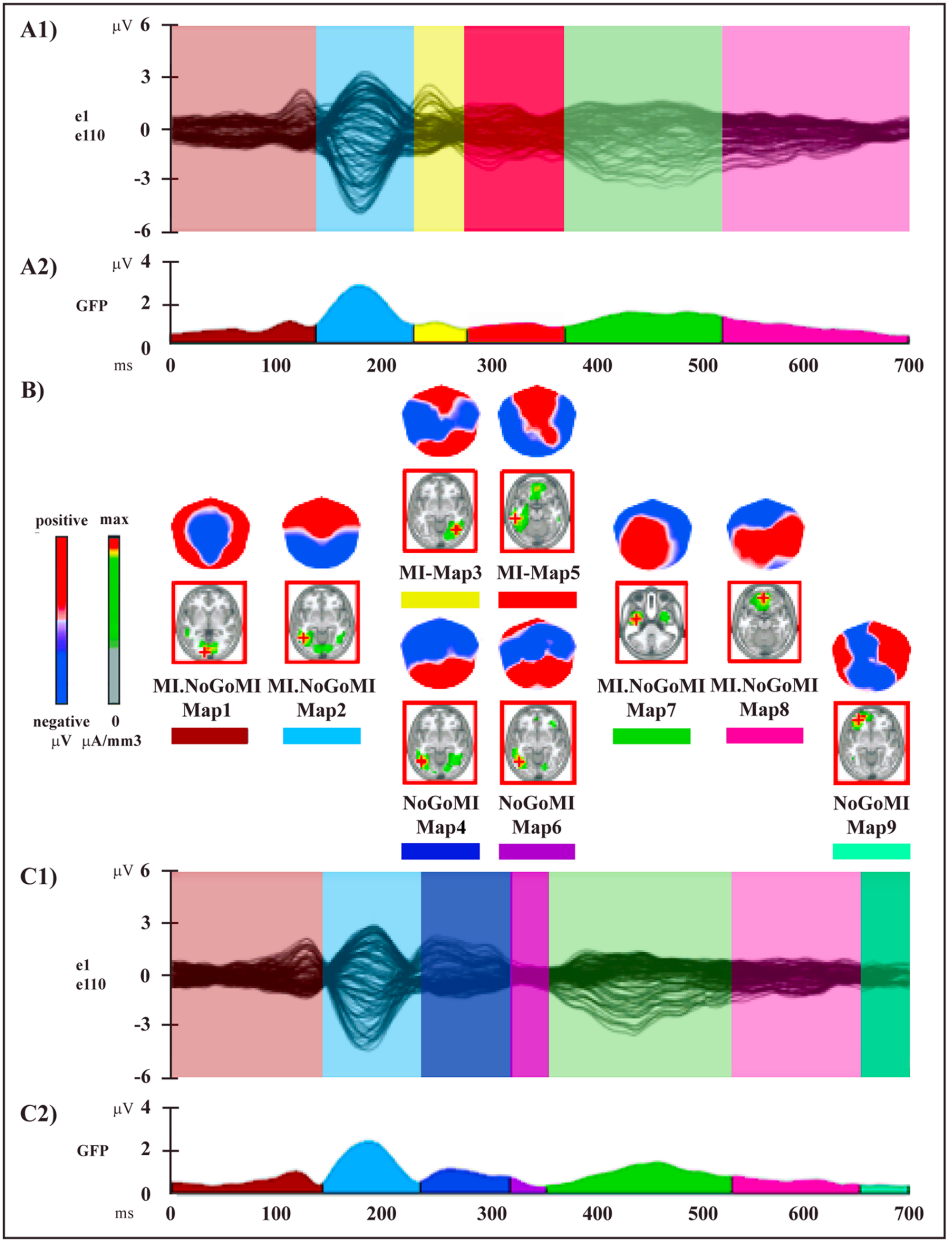
Electrophysiological results over the 700 ms post-stimulus period (stimulus onset: 0 ms) of session B. **(A1** and **C1)** Group-averaged (n = 15) ERP waveforms for Motor Imagery (MI) (A1) and NoGo Motor Imagery (NoGoMI) (C1) conditions, superimposed across the 110 recording channels (e1–e110). **(A2** and **C2)** Microstate segmentation results for MI (A2) and NoGoMI (C2) conditions. **(B)** Mean topographic maps and related mean LAURA source estimations (in red panels) corresponding to each microstate for group-averaged ERP data. All other conventions as in Fig 3.

The two topographic pattern analyses revealed a series of 12 different segmentation maps (i.e., microstates) accounting for the electric field configuration of the collective group-averaged dataset in session A (Go and NoGo conditions) (Fig 3) and 9 maps for session B (MI and NoGoMI conditions) (Fig 4). These two sequences of maps explained respectively 91.89% (session A) and 89% (session B) of the variance in ERPs. Results for each session are presented separately.

#### 1) Session A: Go and NoGo Conditions

The microstate analysis revealed a sequence of 8 maps for the Go condition (Fig 3A1, 3A2 and 3B, Table 1) and of 7 maps for the NoGo condition (Fig 3B,3C1 and 3C2, Table 1). The onset and offset time of each microstate in each condition, resulting from the segmentation analysis of group-averaged data, are reported in Table 1.

**Table 1:** Results of the microstate analysis of session A (Go and NoGo conditions).

Microstate	Onset-offset time (ms): Go	Onset-offset time (ms): NoGo	Talairach coordinates (x,y,z mm)	Brain region label
Go.NoGo-Map 1	0–108	0–112	48,-48,-13	Right fusiform gyrus, BA^1^ 37
Go.NoGo-Map 2	110–140	114–144	48,-48,-13	Right fusiform gyrus, BA 37
Go.NoGo-Map 3	142–214	146–220	-48,-55,-6	Left middle occipital gyrus, BA 19
Go-Map 4	216–254		41,-62,-6	Right fusiform gyrus, BA 37
NoGo-Map 5		222–316	48,-55,-6	Right middle occipital gyrus, BA 19
Go-Map 6	256–304		56,-41,-13	Right middle temporal gyrus, BA 20
Go-Map 7	306–410		-48,-12,-27	Left inferior temporal gyrus, BA 20
NoGo-Map 8		318–432	33,1,-35	Right inferior temporal gyrus, BA 20
Go-Map 9	412–546		-41,-62,-6	Left fusiform gyrus, BA 37
NoGo-Map 10		434–536	33,1,-35	Right inferior temporal gyrus, BA 20
NoGo-Map 11		538–700	41,1,-35	Right middle temporal gyrus, BA 38
Go-Map 12	548–700		33,47,-11	Right middle frontal gyrus, BA 11

^1^BA: Brodmann Area

Onset and offset time (in ms post-target onset) of each microstate in each condition resulting from segmentation analysis applied to group-averaged session A dataset are shown, with Talairach and Tournoux coordinates and corresponding brain region label of maximum of current source density of each mean template map.

In particular, while 3 maps (Go.NoGo-Maps 1, 2 and 3) were found in both conditions, different maps were observed between Go and NoGo conditions over the 216–316 ms time period (Go-Maps 4 and 6, NoGo-Map 5) and the 318–700 ms time period (Go-Maps 7, 9, 12; NoGo-Maps 8, 10, 11): these intervals correspond to the NoGo-N2 and NoGo-P3 latencies of ERP waveform components (see Fig 2).

The reliability of these microstates was assessed at the individual level by means of the fitting procedure (see: Materials and Methods), applied in three time windows based on the appearance of maps in the group-averaged segmentation results (Fig 3A2 and 3C2, Table 1). Since the fitting procedure implies the preselection of time windows of equal duration between conditions, results of the ANOVAs on number of time frames for the main effect of Condition were always not significant; hence they will not be reported.

In the first time window (0–220 ms), Go.NoGo-Maps 1, 2 and 3 were included in the fitting. The 2 x 3 ANOVA did not yield significant results (main effect of Map: F _(2,28)_ = 3.09, P > 0.05; Condition x Map interaction: F _(2,28)_ = 1.5, P > 0.05), in accord with the segmentation data showing the same map sequence in the two conditions.

In the second time window (216–316 ms), Go-Maps 4, 6 and NoGo-Map 5 were included in the fitting. The 2 x 3 ANOVA showed a significant Condition x Map interaction (F _(2,28)_ = 19.21, P < 0.0001): this result indicated that different maps better accounted for each condition, as confirmed by planned comparisons for Go-Map 6 (F _(1,14)_ = 19.9, P = 0.0005) and for NoGo-Map 5 (F _(1,14)_ = 29.36, P < 0.0001), but not for Go-Map 4 (F _(1,14)_ = 1,68, P > 0.05).

In the third time window (306–700 ms), Go-Maps 7, 9, 12 and NoGo-Maps 8, 10, 11 were included in the fitting; the 2 x 6 ANOVA showed a significant main effect for Map (F _(5,70)_ = 5.53, P < 0.0005), due to the different durations of the various microstates (Fig 3A2 and 3C2); more importantly, Condition x Map interaction was significant (F _(5,70)_ = 8.49, P < 0.0001); planned comparisons confirmed the significant difference of map presence between conditions for all maps, except for NoGo-Map 11 (Go-Map 7: F _(1,14)_ = 15.18, P < 0.005; Go-Map 9: F _(1,14)_ = 6.91, P < 0.05; Go-Map 12: F _(1,14)_ = 6.36, P < 0.05; NoGo-Map 8: F _(1,14)_ = 16.08, P < 0.005; NoGo-Map 10: F _(1,14)_ = 7.78, P < 0.05; NoGo-Map 11: F _(1,14)_ = 3.42, P > 0.05).

In summary, for all maps, except for Go-Map 4 and NoGo-Map 11, the fitting procedure confirmed at the single-subject level the segmentation results obtained at the group-averaged level for Go and NoGo conditions.

#### 2) Session B: Motor Imagery and NoGo Motor Imagery Conditions

The microstate analysis revealed a sequence of 6 maps for the MI condition (Fig 4A1, 4A2 and 4B, Table 2) and of 7 maps for the NoGoMI condition (Fig 4B, 4C1 and 4C2, Table 2). The onset and offset time of each microstate in each condition, resulting from the segmentation analysis of group-averaged data, are reported in Table 2.

**Table 2:** Results of the microstate analysis of session B (MI and NoGoMI conditions).

Microstate	Onset-offset time (ms): MI	Onset-offset time (ms): NoGoMI	Talairach coordinates (x,y,z mm)	Brain region label
MI.NoGoMI-Map 1	0–144	0–148	-11,-91,1	Left lingual gyrus, BA^1^ 17
MI.NoGoMI-Map 2	146–224	150–228	-48,-55,-6	Left middle occipital gyrus, BA 19
MI-Map 3	226–268		41,-62,-6	Right fusiform gyrus, BA 37
NoGoMI-Map 4		230–306	-48,-62,-6	Left middle occipital gyrus, BA 37
MI-Map 5	270–356		-56,-33,-14	Left inferior temporal gyrus, BA 20
NoGoMI-Map 6		308–346	-48,-62,-6	Left middle occipital gyrus, BA 37
MI.NoGoMI-Map 7	358–500	348–514	-41,-5,-34	Left middle temporal gyrus, BA 21
MI.NoGoMI-Map 8	502–700	516–644	-3,46,-18	Left medial frontal gyrus, BA 11
NoGoMI-Map 9		646–700	-33,47,-11	Left middle frontal gyrus, BA 11

^1^BA: Brodmann Area

Onset and offset time (in ms post-target onset) of each microstate in each condition resulting from segmentation analysis applied to group-averaged session B dataset are shown, with Talairach and Tournoux coordinates and corresponding brain region label of maximum of current source density of each mean template map.

The two conditions were characterized by the presence of different segmentation maps from 226 to 356 ms (MI-Maps 3, 5 and NoGoMI-Maps 4, 6) and from 646 to 700 ms (MI.NoGoMI-Map 8 and NoGoMI-Map 9) after target onset. The same sequence of common topographical maps appeared in both conditions in the remaining period (Fig 4).

The reliability of these microstates was assessed at the individual level by means of the fitting procedure, applied in three time windows based on the appearance of maps in group-averaged segmentation results (Fig 4A2 and 4C2, Table 2).

In the first time window (0–228 ms) MI.NoGoMI-Maps 1 and 2 were included in the fitting; the 2 x 2 ANOVA did not yield significant results (main effect of Map: F _(1,14)_ = 0.35, P > 0.05; Condition x Map interaction: F _(1,14)_ = 0.25, P > 0.05), in accord with the segmentation data, showing the same map sequence in the two conditions.

In the second time window (226–356 ms) MI-Maps 3 and 5 and NoGoMI-Maps 4 and 6 were fitted; the 2 x 4 ANOVA showed a significant main effect of Map (F _(3,42)_ = 14.56, P < 0.0001) due to different duration of the various maps (Fig 4A2 and 4C2) and, more importantly, a significant Condition x Map interaction (F _(3,42)_ = 10.14, P < 0.0001). Planned comparisons confirmed the significant difference of map presence between the two conditions for MI-Map 5 (F _(1,14)_ = 11.56, P < 0.005) and for NoGoMI-Map 4 (F _(1,14)_ = 11.8, P < 0.005), but not for MI-Map 3 (F _(1,14)_ = 0.007, P > 0.05) and for NoGoMI-Map 6 (F _(1,14)_ = 3.47, P > 0.05).

In the third time window (348–700 ms) MI.NoGoMI-Maps 7 and 8 and NoGoMI-Map 9 were included in the fitting; the 2 x 3 ANOVA showed a significant main effect for Map (F _(2,28)_ = 10.53, P < 0.005), due to the different duration of the maps (Fig 4), but did not show significant Condition x Map interaction (F _(2,28)_ = 1.3, P > 0.05), confirming that the segmentation maps did not differed between the two conditions.

In summary, for all maps, except for MI-Map 3 and NoGoMI-Maps 6 and 9, the fitting procedure confirmed at the single-subject level the segmentation results obtained at the group-averaged level for MI and NoGoMI conditions.

### Source Analysis

The results of the group-averaged LAURA source estimations of each mean map of the four conditions are shown in Figs 3B and 4B and the Talairach and Tournoux coordinates of the current density maximum of each map are summarized in Tables 1 and 2.

Results of the voxel-wise parametric mapping analysis of the sources of the condition-specific microstates statistically confirmed by the fitting procedure will be presented. Areas with significantly different activations (P < 0.05, t _(14)_ > 2.14 / < -2.14; cluster threshold of 10 contiguous activated solution points) will be reported, with t and P-values, Talairach and Tournoux coordinates (x,y,z mm) and anatomical labels of solution points with the local maximum different activities.

#### 1) Session A: Go and NoGo Conditions

**Fig 5:**
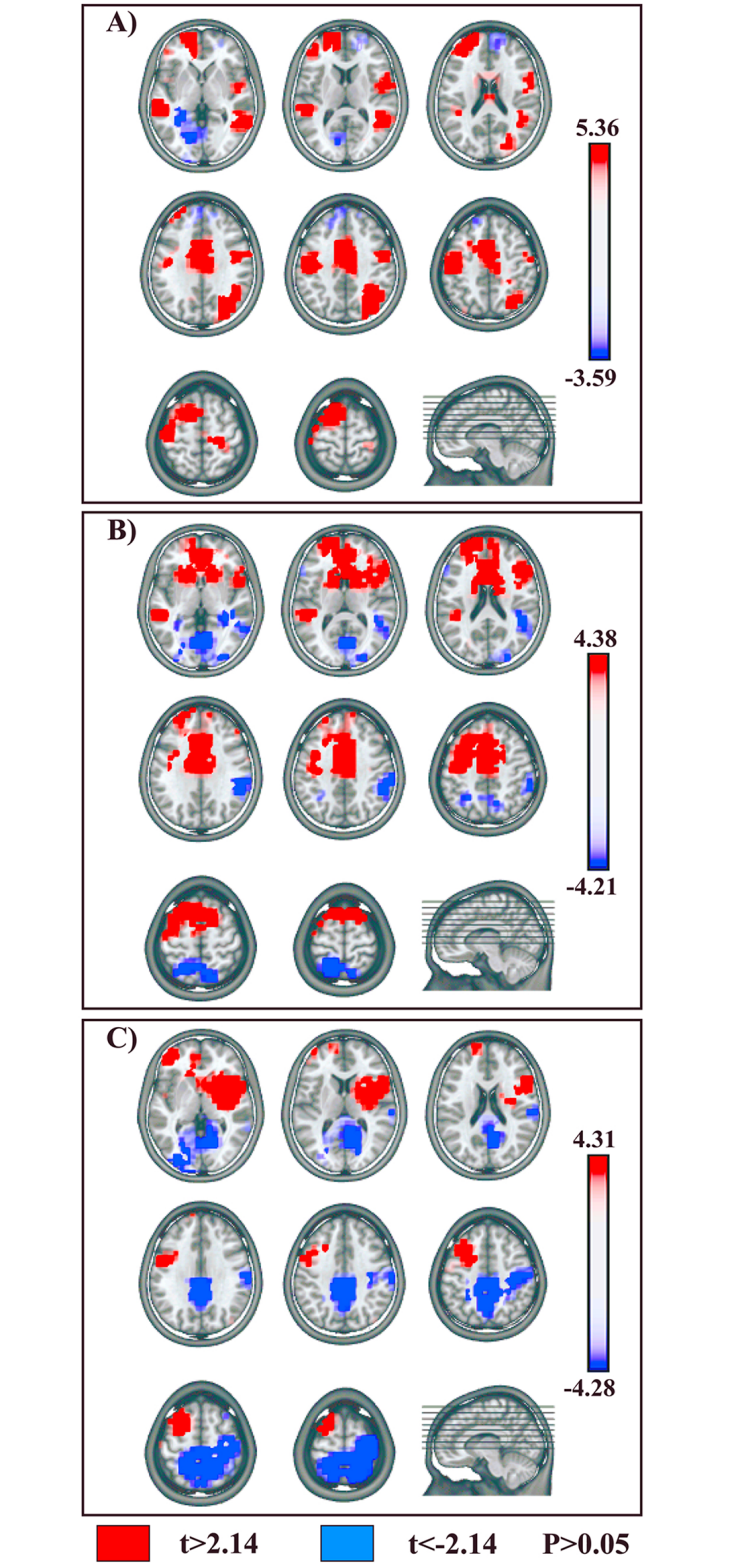
Statistical comparisons of LAURA source estimations between condition-specific microstates. **NoGo vs. Go conditions. (A)** NoGo-Map 5 vs. Go-Map 6. (**B)** NoGo-Map 8 vs. Go-Map 7. **(C)** NoGo-Map 10 vs. Go-Map 9. All significant voxels are colored (t _(14)_ > 2.14 / < -2.14, P < 0.05): positive t-values (red color) indicate higher current source densities in NoGo than in Go condition; negative t-values (blue color) indicate higher current source densities in Go than in NoGo condition. LAURA solutions are rendered on MNI152 template brain.

Voxel-wise paired t-test between NoGo-Map 5 and Go-Map 6 revealed a significant higher activity in NoGo as compared with Go condition (Fig 5A, red) in 5 cortical clusters, localized in: 1) left prefrontal cortex, encompassing fronto-polar cortex (Brodmann Area, BA, 10) and extending toward the DLPFC in middle frontal gyrus (BA 46) (t _(14)_ = 3.49, P < 0.005; -18,63,14; left superior frontal gyrus, BA 10); 2) left pre-SMA (BA 6) and underlying bilateral midcingulate cortex (MCC) (BAs 24, 32) (t _(14)_ = 3.29, P < 0.01; -11,6,51; left medial frontal gyrus, BA 6); 3) left dPMC, encompassing left middle frontal and adjacent precentral gyrus (BA 6) (t _(14)_ = 2.59, P < 0.05; -26,13,58; left middle frontal gyrus, BA 6); 4) right IPL (BAs 39, 40) (t _(14)_ = 5.04, P < 0.0005; 33,-53,34; right IPL, BA 40); 5) left middle and superior temporal gyri (BA 22) (t _(14)_ = 5.36, P = 0.0001; -56,-32,5; left middle temporal gyrus, BA 22).

Higher activity in Go as compared to NoGo condition (Fig 5A, blue) was found in left temporo-occipital areas, encompassing inferior temporal and fusiform gyrus (BAs 20, 37) (t _(14)_ = -3.59, P < 0.005; -33,-33,-14; left temporal fusiform gyrus, BA 20).

The voxel-wise paired t-test comparing NoGo-Map 8 and Go-Map 7 showed a significantly higher activation in NoGo condition (Fig 5B, red) in 4 anterior cerebral clusters localized in: 1) left fronto-polar cortex (BA 10) (t _(14)_ = 2.75, P < 0.05; -18,63,14; left superior frontal gyrus, BA 10); 2) bilateral pre-SMA (BA 6) and underlying MCC, extending anteriorly in perigenual ACC (BAs 24, 32) (t _(14)_ = 4.38, P < 0.001; 3,13,44; right medial frontal gyrus, BA 6); 3) left dPMC (BA 6) (t _(14)_ = 3.28, P < 0.01; -33,1,38; left middle frontal gyrus, BA 6); 4) right IFG (BA 45) (t _(14)_ = 2.71, P < 0.05; 41,19,16; right IFG, BA 45).

Higher activations in Go condition (Fig 5B, blue) were found in 4 posterior cerebral clusters localized in: 1) right IPL (BA 40) (t _(14)_ = -3.44, P < 0.005; 56,-38,33; right supramarginal gyrus, BA 40); 2) left superior parietal lobule (SPL) (BA 7) (t _(14)_ = -2.65, P < 0.05; -18,-51,61; left SPL, BA 7); 3) bilateral occipital extrastriate visual areas, including cuneus, occipital middle, inferior and lingual gyri (BA 18) (t _(14)_ = -4.21, P < 0.001; 3,-69,0; right lingual gyrus, BA 18); 4) left temporo-occipital areas, encompassing inferior temporal and fusiform gyri (BAs 20, 37) (t _(14)_ = -3.08, P < 0.01; -56,-12,-21; left inferior temporal gyrus, BA 20).

The voxel-wise t-test comparing NoGo-Map 10 and Go-Map 9, revealed stronger activations in NoGo condition (Fig 5C, red) in 4 cerebral clusters in: 1) left fronto-polar cortex (BA 10) (t _(14)_ = 3.69, P < 0.005; -41,47,-5; left middle frontal gyrus, BA 10); 2) left dPMC (BA 6) (t _(14)_ = 2.89, P < 0.05; -33,13,44; left middle frontal gyrus, BA 6); 3) right IFG (BAs 44, 45, 47) and anterior insula (BA 13) (t _(14)_ = 4.31, P < 0.001; 41,4,10; right insula, BA 13); 4) left middle temporal gyrus (BA 21) (t _(14)_ = 2.73, P < 0.05; -56,3,-9; left middle temporal gyrus, BA 21).

Enhanced activity in Go condition (Fig 5C, blue) was found in 2 posterior cerebral clusters localized in: 1) bilateral SPL and precuneus (BA 7), extending on the right side toward postcentral gyrus (BAs 3, 5) (t _(14)_ = -4.28, P < 0.001; -11,-52,47; left precuneus, BA 7); 2) bilateral occipital extrastriate visual areas in left occipital middle, inferior and lingual gyri and in right cuneus and lingual gyri (BAs 18, 19, 30) (t _(14)_ = -3.97, P < 0.005; -26,-84,1; left middle occipital gyrus, BA 18).

#### 2) Session B: Motor Imagery and NoGo Motor Imagery Conditions

**Fig 6:**
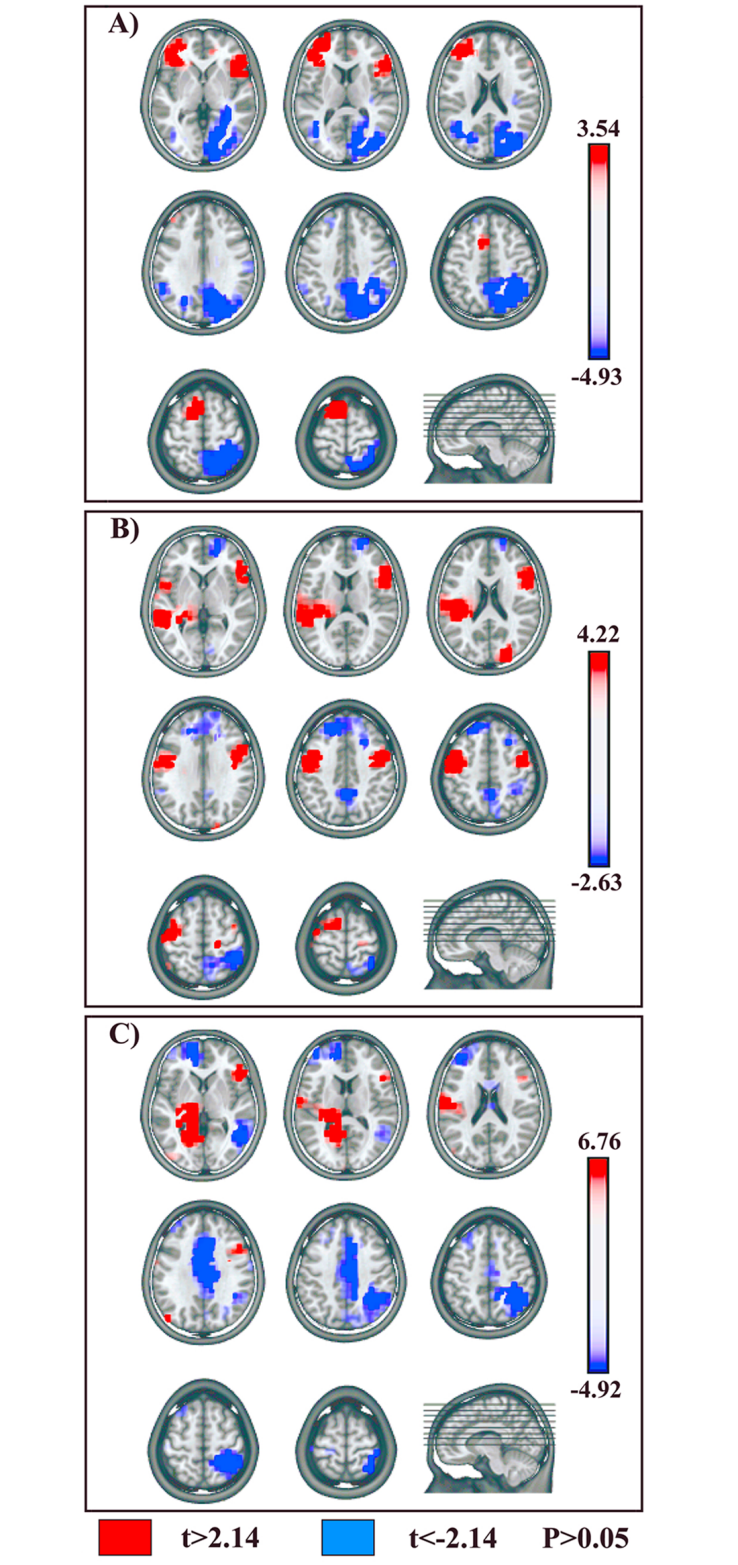
Statistical comparisons of LAURA source estimations between condition-specific microstates. **MI vs.: NoGoMI, Go, NoGo conditions**. **(A)** MI-Map 5 vs. NoGoMI-Map 4. **(B)** MI-Map 5 vs. Go-Map 6. **(C)** MI-Map 5 vs. NoGo-Map 5. Positive t-values (red color) indicate higher current source densities in MI than in the compared condition; negative t-values (blue color) indicate higher current source densities in the compared condition than in MI condition. All other conventions as in Fig 5.

In our periods of interest the topographical and fitting analyses showed that only two microstates (MI-Map 5 and NoGoMI-Map 4) were significantly different between the two conditions (see Fig 4): the voxel-wise t-test revealed a higher activation in MI as compared to NoGoMI (Fig 6A, red) in 3 frontal clusters localized in: 1) left DLPFC, including middle and inferior frontal gyri (BA 46) (t _(14)_ = -3.14, P < 0.01; -41,40,8; left IFG, BA 46); 2) left pre-SMA (BA 6) (t _(14)_ = -2.94, P < 0.05; -11,6,58; left medial frontal gyrus, BA 6); 3) right IFG (BAs 45, 47) (t _(14)_ = -3.54, P < 0.005; 48,18,2; right IFG, BA 47).

Higher activity in NoGoMI as compared to MI (Fig 6A, blue) was found in: 1) right posterior parietal cortex (PPC), encompassing SPL and precuneus (BA 7) and IPL (BA 40) (t _(14)_ = 4.93, P < 0.0005; 41,-52,54; right SPL, BA 7); 2) right occipital extrastriate visual cortex in occipital superior, middle, inferior and lingual gyri (BAs 18, 19) (t _(14)_ = 3.72, P < 0.005; 41,-83,21; middle occipital gyrus, BA 19); 3) left posterior middle and superior temporal gyri (BA 39) (t _(14)_ = 2.79, P < 0.05; -41,-61,20; left middle temporal gyrus, BA 39).

#### 3) Statistical Source Comparison between Sessions A and B

In session B significant topographic differences between conditions were present between 226 and 356 ms post-target onset: this finding suggests that neural activities related to putative motor and inhibitory mechanisms during MI were likely implemented in such time window. Hence, in order to identify differences and/or similarities between supposed inhibitory mechanisms activated during MI and NoGo conditions, we compared MI-Map 5 with microstates evidenced in session A conditions during an overlapping time period, namely Go-Map 6 and NoGo-Map 5 (Figs 3 and 4).

A voxel-wise paired t-test between MI-Map 5 and Go-Map 6 revealed significant higher activity in MI with respect to Go condition (Fig 6B, red) in 4 cerebral clusters localized in: 1) left dPMC encompassing left middle frontal and precentral gyri (BA 6) (t _(14)_ = 4.22, P < 0.001; -48,-1,45; left precentral gyrus, BA 6); 2) right IFG (BAs 44, 45, 47) (t _(14)_ = 2.97, P < 0.05; 56,32,-4; right IFG, BA 47); 3) left middle and superior temporal gyri (BA 22) (t _(14)_ = 3.69, P < 0.005; -48,-40,5; left middle temporal gyrus, BA 22); 4) right anterior middle temporal gyrus (BA 21) and temporo-polar cortex (BA 38) (t _(14)_ = 3.52, P < 0.005; 56,2,-22, right middle temporal gyrus, BA 21).

A voxel-wise t-test comparing MI-Map 5 and NoGo-Map 5 revealed a higher activity in MI (Fig 6C, red) in 2 cerebral clusters in: 1) right IFG (BAs 45, 47) (t _(14)_ = 3.96, P < 0.005; 56,32,-4; right IFG, BA 47); 2) left temporo-occipital areas encompassing inferior temporal and fusiform gyri (BAs 20, 37) (t _(14)_ = 6.76, P < 0.0001; -26,-48,-7; left fusiform gyrus, BA 37).

Higher activity in NoGo condition with respect to MI condition (Fig 6C, blue) was found in: 1) left fronto-polar cortex (BA 10) (t _(14)_ = -3.46, P < 0.005; -18,47,-5; left medial frontal gyrus, BA 10); 2) bilateral MCC (BAs 32, 24) (t _(14)_ = -4.4, P < 0.001; 11,19,30; right cingulate gyrus, BA 32); 3) right PPC, encompassing both SPL (BA 7) and IPL (BA 40), extending toward postcentral gyrus (BAs 3, 5) (t _(14)_ = -4.92, P < 0.0005; 26,-45,54; right SPL, BA 7); 4) right posterior middle temporal gyrus (BAs 21, 37) (t _(14)_ = -2.49, P < 0.05; 56,-62,0; right middle temporal gyrus, BA 37).

## Discussion

The principal aim of the present study was the evaluation of the putative inhibitory mechanisms activated during the covert action of MI, and to compare them with inhibitory control mechanisms of overt actions elicited during an overt NoGo condition. The segmentation analyses revealed the presence of different cerebral microstates, indexing different neural processing and generators [30], both in NoGo with respect to Go, and in MI with respect to NoGoMI conditions: of note, a different temporal distribution of these condition-specific neural activations emerged in the two sessions (Figs 3 and 4). In session A, a sequence of statistically significant microstates different between Go and NoGo conditions started around 220 ms and continued until about 550 ms post-target onset; conversely, in session B, condition-specific microstates, expected to reflect in MI the putative processes of the voluntary rehearsal and of the concomitant inhibition of motor programs, were contained in a time window around 230–360 ms post-target onset. Critically, statistical source analyses comparing microstates different among conditions in these time windows, revealed the activation in both NoGo and MI conditions of the main foci of motor inhibitory control, namely of pre-SMA and rIFG, but with dissimilar timing and patterns of modulation. These results provide new evidence that basic nodes of an inhibitory network are shared in overt and covert actions, and at the same time underscore a different functional interaction of these areas during the two motor performance modalities.

We will discuss our findings regarding inhibition in MI condition in the light of the functional interpretation of the activities emerged during the overt Go/NoGo task. Indeed, our source estimation results could also contribute to clarify processes and related neural substrates activated during time periods overlapping with NoGo-N2 and NoGo-P3, which have been related to motor inhibition of overt actions in Go/NoGo tasks, but which to date are still highly debated.

In Go/NoGo tasks, inhibitory processes are difficult to disentangle from overlapping operations related to executive control: indeed, inhibition in such tasks could be contextualized in terms of a goal-driven response selection, considering the NoGo condition as a form of active voluntary response [44]. Accordingly, our analyses showed that during the overt Go/NoGo task, motor inhibitory control was integrated in the framework of a perceptual decision-making process, and that it was built up in two steps: an early “decisional” phase, in the 220–300 ms post-target onset (overlapping with NoGo-N2 time range, Fig 2), representing the selection of the NoGo response option and the triggering of the inhibitory process, and a subsequent “implementational” phase in which the inhibition was enacted and maintained, in the time range of NoGo-P3 (Fig 2).

Statistical source comparison in session A between condition-specific microstates of the early decisional phase showed simultaneous activity in several brain areas, suggesting different concomitant cerebral operations. In particular, comparing NoGo-Map 5 and Go-Map 6 we found stronger activity in NoGo condition in left prefrontal cortex, left dPMC, left pre-SMA and right IPL. These sources likely represent the tight integration between fronto-parietal circuits, engaged in visuo-motor transformations for the representation of motor response options, and high level prefrontal areas, providing parallel top-down signals biasing the final selection of the correct inhibitory response [45]. In this context, the left DLPFC would retrieve working memory information about task goals and contingencies, providing top-down guidance to response-selection operations ongoing in fronto-parietal areas [46]. In particular, the contribution of the DLPFC would be necessary to successful response inhibition in situations with increased cognitive demand [47], as in the cued CPT type of Go/NoGo task used in the present study. At the same time, the right IPL, through reciprocal interactions with prefrontal cortex and dPMC, would both provide and maintain selected representations of stimulus-response associations and participate in attentional reorienting to behaviorally relevant stimuli [48], focusing cognitive resources at the presentation of the NoGo target. Of note, it has been proposed a role of the right PPC in situations of response conflict between action plans, and in particular in the presence of competition between stimulus-driven action representations and voluntary control of behavior [49; for review see:^50^]. Likely, the higher activity in NoGo with respect to Go condition in left dPMC during the early decisional phase could be inscribed in this perspective. Left dPMC plays a pivotal role in conditional motor behavior, in which response selection relies on arbitrary visuo-motor associations [51, 52]. This area would encode prelearned stimulus-response associations and provide such predictive information to other reciprocally connected nodes of sensory-motor system, such as the PPC, during the goal-oriented response selection [45]. In this regard, it has been shown that also NoGo stimuli can automatically trigger task-response representations [53], which possibly could be usefully integrated during response elaboration, but which also would require active inhibition to avoid overt unwanted movements. An intriguing hypothesis is that dPMC could encode both response-specific motor programs and their concomitant inhibition, in an intrinsic bottom-up loop: this putative form of reactive automatic inhibition resembles the proposed “impulse control” mechanism [54], aimed at the inhibition of the selected motor program during preparation of a delayed response, and representing: “…a self-contained process (…) where the activation of a response representation automatically triggers a corresponding inhibitory tag” [54]. To date, ample evidence sustains the role of left dPMC in inhibition of overt actions: activation in this area has been previously reported in NoGo condition in EEG [e.g., 20] and fMRI studies [e.g., 55], and also in single-unit neuronal recording in monkeys during a countermanding reaching task [56]. In our study, such putative inhibitory activity of left dPMC was sustained during NoGo condition, from about 220 to 535 ms, possibly with a dual function. In the early decisional phase of inhibitory control, it would have favored the selection of NoGo response option, providing a direct inhibition of motor response programs automatically triggered at stimuli presentation; later on, its sustained activity would have contributed to the effective enactment of the selected NoGo decision, during the implementational phase of inhibition. Furthermore, left dPMC engagement in MI condition emerged by contrasting MI-Map 5 and Go-Map 6: the analogous enhanced activity in this area in both NoGo and MI with respect to Go condition, points to its role in motor inhibition in both overt and covert actions, but likely with a specific task-dependent degree or pattern of engagement, according to different strength or type of inhibition required in covert and overt motor modalities. The putative automatic loop of activation-inhibition of motor representations coded by left dPMC, during MI could have contributed to both the voluntary rehearsal and concurrent inhibition of the covert action. Nevertheless, its role would be more relevant in NoGo condition, which requests additional inhibitory resources: indeed, in session A the risk that stimulus-elicited motor representations could reach the threshold for triggering undesired overt responses was higher as compared to session B, because in the former participants were primed to the possibility to make an overt response, while in the latter just a covert action was involved.

Of note, dPMC is one of the so called “negative motor areas” (NMAs), i.e. cortical regions whose electrical stimulation induces the inability to perform voluntary movements or sustained muscle contraction, without muscular weakness [57; for review see: 58]. Classically, the two main NMAs were identified in correspondence of the IFG (“primary NMA”) and of the pre-SMA (“supplementary NMA”) [58], which also represent the principal nodes of the hypothesized motor inhibitory network, and whose activity emerged during NoGo and MI conditions in our paradigm. In particular, in the overt Go/NoGo task higher activity in pre-SMA emerged in NoGo-Map 5 with respect to Go-Map 6, during the early phase of inhibition. Due to its large connection with prefrontal, PPC and other premotor areas [59], pre-SMA is optimally situated to transform information about the appropriateness of the response options elaborated in parieto-premotor circuits and prefrontal regions, into the selection or preparation of the correct response. To date, the specific role of pre-SMA in motor control is still debated, as this area has been implicated in a wide range of functions, including selection of actions (either overt or covert ones), switching between action plans and motor inhibition [44, 60]. This multiplicity of putative roles has been summarized in one fundamental function: the resolution of the competition within a contingent set of alternative response options, whose neural representations could be activated by external stimuli as well as by internal biases [60, 61]. Pre-SMA would enact an inhibitory mechanism aimed at the suppression of motor representations of unwanted responses to favor selection of the most appropriate one [61, 62]: the motor inhibition of NoGo condition would just represent a particular instantiation of this general pre-SMA activity. It has been proposed that the inhibitory activity of pre-SMA occurs within a network including the rIFG and BG [7, 9], but to date, the temporal hierarchy of activation of these regions is still unclear [63, 64]. In our study, the high temporal resolution of EEG technique allowed us to define the sequential engagement of these areas during our overt Go/NoGo task, namely in the pre-SMA first and in the rIFG subsequently. Indeed, a higher activation in rIFG emerged in NoGo-Map 8 with respect to Go-Map 7. Critically, NoGo-Map 8 was comprised in the 318–432 ms post-target onset: the higher rIFG activity during this microstate could effectively reflect a real-time motor inhibitory mechanism, as it started about 100 ms before the mean Go EMG onset (415 ± 69 ms post-Go target onset).

A new crucial finding of our study is the activation during MI of the main foci of the hypothesized circuit underpinning inhibition of overt actions (namely, the pre-SMA and rIFG), which emerged by statistical source comparison between MI-Map 5 and NoGoMI-Map 4.

Theoretically, alternative explanations for pre-SMA and rIFG activations in NoGo and in MI conditions could be argued [e.g., 19, 65, 66]: in particular, they could be accounted for by different levels of conflict or of cognitive and attentional load between conditions, due to a higher frequency of Go/MI with respect to NoGo/NoGoMI targets. In fact, our data rule out such alternative hypotheses. Indeed, the influence of the “categorical probability” (related to the class to which stimuli are assigned by task instructions) [14] can be excluded, since Go and NoGo trials in session A and MI and NoGoMI trials in session B had equal frequency. Moreover, also the potential influence of the “single stimulus probability” [14] (i.e., higher level of conflict or cognitive and attentional effort needed to individuate each of the 10 different infrequent “noX” letters used as NoGo and NoGoMI targets, with respect to the more frequent “X” letter, representing Go and MI targets) can be excluded. Indeed, higher activity in pre-SMA and rIFG were found in conditions instructed by targets with different “single stimulus probability”, since the 10 infrequent “noX” letters instructed the NoGo condition and conversely the frequent “X” letter instructed the MI condition.

Our data further extend the previously proposed similarities between the neural substrates of covert and overt actions [2] also in the context of the cerebral mechanisms underpinning their motor inhibition. Nonetheless, at the same time important divergences in the inhibition of overt and covert motor performance emerged, suggesting different patterns of temporal recruitment of inhibitory areas, tuned with the overt or covert motor context and with the intended final task goal. Indeed, the inhibitory control of the overt action in NoGo condition sequentially developed in early pre-SMA-related decisional phase and late rIFG-related implemention phase; on the contrary, during MI, inhibition was carried out in a single step, with the concomitant engagement of pre-SMA and rIFG. Hence, the inhibition of the rehearsed motor programs during MI appeared strictly intertwined with response-selection operations: this sort of pre-wired coupling between these two processes suggests that an inhibitory mechanism related to the rIFG might have been *a priori* integrated into the process of selection and voluntary rehearsal of movement representations, as an intrinsic component of the MI enactment.

Of note, MI could be viewed as a particular type of covert action in which the movement representation is voluntarily rehearsed and concurrently automatically inhibited. Inhibition during MI could be considered “automatic” since it runs to completion autonomously, without volitional effort [67]. Nevertheless, although when individuals imagine they don’t deliberately think to put into effect inhibitory commands *per se*, they are aware that they will not overtly move: hence, motor inhibition during MI could yet be included in a goal-oriented “covert modality” of motor performance. This would represent a form of “contingent” or “conditional” automaticity [67, 68], wherein a cerebral process, even if triggered and implemented automatically, is still conditioned on contingently activated top-down goals. In line with hypothesis, previous studies [69, 70] demonstrated that cerebral foci for the controlled inhibition of overt actions, such as the pre-SMA and rIFG, can be triggered unconsciously but yet with a “contingent” automaticity, depending on the presence of a specific activated executive set [71]. Indeed, it has been shown that rIFG activity can be automatically triggered by stimuli that were previously associated with stopping, without the requirement of actual top-down controlled motor inhibition [69]. Moreover, it has been demonstrated that an unconscious, strongly masked NoGo stimulus can activate the pre-SMA and IFG [70]. In our results, the partial overlap in cerebral nodes underpinning the controlled inhibition of overt actions in NoGo condition and the automatic inhibition of covert actions in MI condition, is consistent with the growing literature [for reviews see: 72–74] that questions the traditional dichotomy between automatic (i.e., implicit, outside the phenomenal awareness, conscious intention and volitional effort) and controlled (i.e., explicit, conscious, voluntary and cognitively effortful) cerebral processes [75, 76]: our findings further suggest that automatic and unconscious motor control processes can form an intrinsic part of all voluntary, goal-oriented behaviors [50, 77].

Possibly, in our study during session B, the instructed covert performance modality itself could have intrinsically predisposed the rIFG activation in response to the MI target, allowing an automatic but still goal-oriented inhibition to be implemented during the voluntary rehearsal of motor representations. This view is in accord with the results of a previous EEG study [78] that revealed the influence on information processing of the anticipated overt and covert motor modalities, not only at a late stage of motor performance enactment, but already at an early stage of stimulus processing. Our data further extend these findings in a motor inhibitory perspective: the performance modality of the possible incoming movement, contained in the instructed task goals, likely *ab initio* differentially predisposed an intrinsic reorganization of the parieto-frontal areas designated for sensory-motor transformations and for motor inhibitory control in the two sessions.

Of note, these conclusions go in the direction of a “proactive” control account. In the framework of motor inhibition, two distinct operating strategies have been described: “proactive” and “reactive” control modes [9]. While reactive inhibition is phasically enacted after the detection of the inhibitory signal, in the proactive modality inhibitory circuits could be primed by predictive cues in preparation for the upcoming inhibition [9, 79] without being effectively implemented: this would create a “proactive inhibitory set” [9, 79] through different cortical-BG circuits, allowing inhibition to be more quickly triggered at the presentation of inhibitory signals. Accordingly, it has been shown that proactive and reactive inhibition engage partially overlapping cerebral networks, including the pre-SMA, rIFG, IPL and the BG [79–82]. Nonetheless, to date exact mechanisms and functional meaning of proactive inhibition have yet to be clarified [e.g., for different hypotheses see: 9, 83]. In our CPT, the “O” cue could have primed the motor inhibitory circuit or its parts, favoring the reactive triggering of the inhibitory control when required, namely, at NoGo target onset for the controlled inhibition of overt actions, and at MI target onset, for automatic inhibition of ongoing motor representations. With regard to the latter condition, the concomitant activation of the pre-SMA and rIFG suggests a primed insertion, into the preselected covert modality of motor performance, of an inhibitory mechanism (likely underpinned by the rIFG), which could be subsequently effectively implemented during MI enactment in a contingent automatic manner. Future research is needed to confirm this hypothesis and to investigate whether the proposed strict cooperation between proactive and reactive inhibition required for a successful motor control of overt actions, could be also relevant in the covert motor context.

## Conclusions

The results presented here make two novel contributions to the current literature on motor inhibitory control. First, we showed that covert actions as MI, automatically engage key nodes of the putative inhibitory circuit activated for the controlled inhibition of overt actions. These findings further extend the proposed similarities of neural substrates of covert and overt actions [2] into the framework of motor inhibition; at the same time, our data underline that functional equivalence between overt and covert actions is only partial, since an inhibitory mechanism could be pre-wired into the covert motor performance modality. Second, our data show that controlled and automatic forms of motor inhibition are implemented by shared basic mechanisms and cerebral substrates, but with different patterns of engagement, in accord with the intended overt or covert motor context.

The evidence that controlled and automatic motor inhibition share partially overlapping basic mechanisms, together with the goal-oriented nature of automatic inhibition during MI, further challenges the rigid dichotomy between conscious, explicit, flexible and unconscious, implicit, inflexible forms of behavioral control.
